# Two Clade A Phosphatase 2Cs Expressed in Guard Cells Physically Interact With Abscisic Acid Signaling Components to Induce Stomatal Closure in Rice

**DOI:** 10.1186/s12284-019-0297-7

**Published:** 2019-05-27

**Authors:** Myung Ki Min, Eun-Hye Choi, Jin-Ae Kim, In Sun Yoon, Seungsu Han, Yeongmok Lee, Sangho Lee, Beom-Gi Kim

**Affiliations:** 1 Metabolic Engineering Division, Department of Agricultural Biotechnology, National Institute of Agricultural Sciences, Rural Development Administration, Jeonju, 54875 Republic of Korea; 2 Gene Engineering Division, Department of Agricultural Biotechnology, National Institute of Agricultural Sciences, Rural Development Administration, Jeonju, 54875 Republic of Korea; 3 Department of Biological Sciences, Sungkyunkwan University, Suwon, 16419 Republic of Korea

**Keywords:** Rice, Guard cell, ABA, Stomata, Phosphatase

## Abstract

**Background:**

The core ABA signaling components functioning in stomatal closure/opening, namely ABA receptors, phosphatases, SnRK2s and SLAC1, are well characterized in Arabidopsis, but their functions in guard cells of rice have not been extensively studied.

**Results:**

In this study, we confirmed that *OsSLAC1*, the rice homolog of *AtSLAC1*, is specifically expressed in rice guard cells. Among the rice *SAPKs*, *SAPK10* was specifically expressed in guard cells. In addition, SAPK10 phosphorylated OsSLAC1 in vitro and transgenic rice overexpressing *SAPK10* or *OsSLAC1* showed significantly less water loss than control. Thus, those might be major positive signaling components to close stomata in rice. We identified that only OsPP2C50 and OsPP2C53 among 9 OsPP2CAs might be related with stomatal closure/opening signaling based on guard cell specific expression and subcellular localization. Transgenic rice overexpressing *OsPP2C50* and *OsPP2C53* showed significantly higher water loss than control. We also characterized the interaction networks between OsPP2C50 and OsPP2C53, SAPK10 and OsSLAC1 and found two interaction pathways among those signaling components: a hierarchical interaction pathway that consisted of OsPP2C50 and OsPP2C53, SAPK10 and OsSLAC1; and a branched interaction pathway wherein OsPP2C50 and OsPP2C53 interacted directly with OsSLAC1.

**Conclusion:**

OsPP2C50 and OsPP2C53 is major negative regulators of ABA signaling regarding stomata closing in rice. Those can regulate the OsSLAC1 directly or indirectly thorough SAPK10.

**Electronic supplementary material:**

The online version of this article (10.1186/s12284-019-0297-7) contains supplementary material, which is available to authorized users.

## Background

In plants, stomatal apertures represent the main gateways for exchange of gases, such as O_2_, CO_2_, and water vapor between the leaf and the atmosphere. Stomata open or close in response to light, CO_2_ concentration, atmospheric humidity, ozone, and small stress-generated signaling molecules such as abscisic acid (ABA), jasmonic acid, Ca^2+^, nitric oxide, and reactive oxygen species (Bauer et al. 2013; Hetherington and Woodward 2003; Kim et al. 2010; Schroeder et al. 2001). The stomatal aperture is a pore surrounded by two guard cells, which can be expanded or shrunk by changes in turgor pressure. Channels such as the K^+^-outward-rectifying channel (GORK), K^+^-inward-rectifying channel (KAT1), rapid-type anion channel and slow-type anion channel (SLAC1) regulate the turgor pressure of guard cells by transporting ions including K^+^, Cl^−^, and malate (Ache et al. 2000; Anderson et al. 1992; Kim et al. 2015; Schachtman et al. 1992; Vahisalu et al. 2008). Among these channels, SLAC1 has been reported to be crucial for closing the stomata in *Arabidopsis thaliana* (Arabidopsis). Activated SLAC1 depolarizes the plasma membrane to activate GORK and push K^+^ out from the guard cell, thereby reducing the turgor pressure and closing the stomatal aperture (Sirichandra et al. 2009).

ABA and Ca^2+^ signaling regulate SLAC1 activity via phosphorylation and dephosphorylation. Serine 120 (Ser120) of Arabidopsis SLAC1 is phosphorylated and activated in the presence of ABA, mainly by OPEN STOMATA 1 (OST1; also called SnRK2E or SnRK2.6), a SNF1-RELATED PROTEIN KINASE2 (SnRK2) family protein (Geiger et al. 2009; Lee et al. 2009; Vahisalu et al. 2010). In addition, CALCIUM-DEPENDENT PROTEIN KINASE 6/21/23 (CPK 6/21/23) and CBL-INTERACTING PROTEIN KINASEs (CIPKs), which play roles in Ca^2+^-dependent signaling, activate SLAC1 by phosphorylating Ser59 in response to Ca^2+^ signaling (Brandt et al. 2012; Geiger et al. 2010; Maierhofer et al. 2014). The clade A type 2C protein phosphatases (PP2CAs) play negative regulatory roles in stomatal closure via ABA and Ca^2+^ signaling (Mustilli et al. 2002; Zhang et al. 2014). PP2CAs suppress ABA signaling by interacting with and inactivating OST1 and CPK6. On other hand, PP2CAs directly interact with and inactivate SLAC1 by dephosphorylation, counteracting its phosphorylation by OST1 or CPKs in response to ABA or Ca^2+^ signaling (Brandt et al. 2012; Geiger et al. 2009; Lee et al. 2009).

The core ABA signaling components, which consist of ABA receptors, PP2CAs and SnRK2s, are well conserved among plants, even though monocots and dicots have different types of guard cells, dumbbell and kidney types, respectively (Hauser et al. 2011; Schafer et al. 2018). Thus, it is necessary to systematically identify and compare the differences and similarities among ABA signaling components functioning in monocot and dicot guard cells. To date, there are few reports regarding core ABA signaling components focused on monocot guard cells (Schafer et al. 2018; Sun et al. 2016). Recently it has been reported that monocot SLAC1s such as OsSLAC1 and HvSLAC1 of rice and barley require extracellular nitrate for activation but dicot SLAC1s do not (Schafer et al. 2018; Sun et al. 2016). However, it was not known whether the molecular mechanisms of ABA signaling are different between Arabidopsis and rice (*Oryza sativa*).

Here, we identified the core ABA signaling components functioning in rice guard cells and characterized their interaction networks. This study provides useful information about the molecular mechanisms of monocot guard cell movement. Furthermore, this information will contribute to improved water usage efficiency in crops through guard cell regulation.

## Results

### *OsSLAC1* is Expressed in Guard Cell and Overexpression Lines of *OsSALC1* Lose Less Water in Rice

**Fig. 1:**
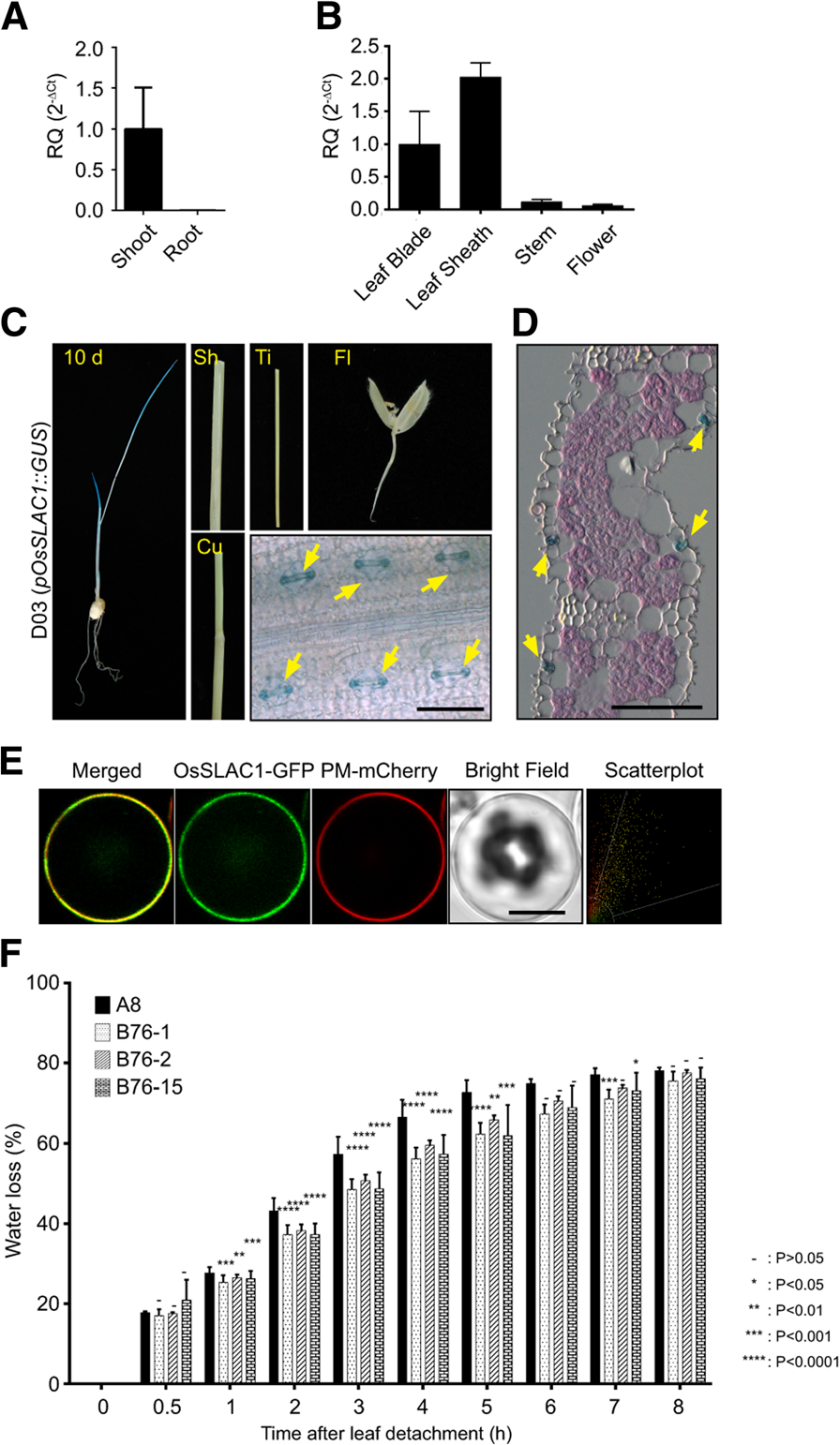
*OsSLAC1* is specifically expressed in rice guard cells and OsSLAC1-GFP localizes to the plasma membrane in rice protoplasts. **a** RT-qPCR analysis of *OsSLAC1* gene expression in two-week-old seedlings. The generated RQ (relative quantity, 2^-ΔCt^) was compared to the value of expression on shoot. *OsUbi5* was used as endogenous control. The values depict the average and ± SD with 3 replications. **b** RT-qPCR analysis of *OsSLAC1* gene expression in different tissues of mature rice (80 days old). The values were compared to the expression level on leaf blade. **c** Histochemical β-glucuronidase analysis of *OsSLAC1* promoter-GUS transgenic rice. Left image is a 10-day-old seedling. Sh (sheath), Ti (tiller), Fl (flower), Cu (culm) and leaf blade epidermis (bottom right). Scale bars are 50 μm. **d** A section of paraffin-embedded leaf blade stained with X-Gluc (blue) and eocin. Yellow arrows indicate guard cells. Scale bars are 50 μm. **e** Subcellular localization of OsSLAC1-GFP and marker protein in rice protoplast. PM-mCherry is a plasma membrane localization marker. The scatterplot shows the values of fluorescence of the pixels across the two channels. Scale bars are 10 μm. **f** Water loss assays of *OsSALC1*-overexpressing transgenic lines (B76–1, 2 and 15) and control line (A8: empty vector transgenic plant). The values depict the average and ± SD with 3 replications. ANOVA analysis was performed by comparing with water loss of A8 plant

The guard cell-expressed *AtSLAC1* functions as an S-type anion channel and is one of the major targets of ABA signaling for stomatal closure in Arabidopsis. Previously, OsSLAC1 (Os04g18530) was identified as the most closely related rice protein to AtSLAC1 (Additional file 1: Figures S1A) (Kusumi et al. 2012). OsSLAC1 has been reported to regulate CO_2_ homeostasis in rice and function as a nitrate-selective anion channel when expressed in Xenopus oocytes (Kusumi et al. 2012; Sun et al. 2016), and to present in rice guard cells confirmed by own promoter GUS expression analysis (Kusumi et al. 2017). We also analyzed gene expression profiles of the rice S-type anion channels in different tissues using the Rice Expression Profile Database (http://ricexpro.dna.affrc.go.jp) and Genevestigator (http://genevestigator.com) (Additional file 1: Figure S1B and Additional file 6: Figure S6A). Among the S-type anion channels of rice, *OsSLAC1* was expressed predominantly in the leaf blade, even though other homologues are expressed in roots or stems. When we examined the expression of *OsSLAC1* using RT-qPCR in two-week-old seedlings, *OsSLAC1* expression was dramatically higher in shoots than in roots (Fig. 1a). When total RNA was isolated from leaf blade, leaf sheath, stem and flower tissues of mature rice (80-day-old plants), *OsSLAC1* expression was found to be higher in leaf tissues (including leaf blade and sheath) than in other tissues (Fig. 1b). To examine the tissue-specific expression of *OsSLAC1* in more detail, we constructed transgenic rice lines (D03) harboring an *OsSLAC1* promoter-β-glucuronidase (*GUS*) reporter vector (*pOsSLAC1::GUS*). Leaves showed strong X-Gluc staining, but roots were not stained in 10-day-old transgenic rice seedlings (Fig. 1c). Mature transgenic rice, including *pOsSLAC1-GUS*, did not express *GUS* in culm, tiller or flower. The epidermis of stained seedling leaves was observed under a microscope and showed GUS staining specifically in guard cells (Fig. 1c). We sectioned stained seedling leaves to dissect the expression in detail and we confirmed that X-Gluc staining was detected only in the guard cells and not in mesophyll cells or other epidermal cells (Fig. 1d). Thus, we can conclude that OsSLAC1 is expressed specifically in guard cell. We also confirmed the subcellular localization of OsSLAC1-GFP. The fluorescence signal of OsSLAC1-GFP overlapped with the signal of a plasma membrane-localized marker (PM-mCherry) in rice protoplasts (Fig. 1e).

To identify the effects of OsSLAC1 in stomatal closure, we constructed transgenic rice lines overexpressing *OsSLAC1* (B76). Three *OsSLAC1* over-expression lines were selected based on gene expression level (Additional file 4: Figure S4A). Transgenic plant lines harboring the empty vector were used as the control and named as A8. Water loss ratios of the *OsSLAC1*-overexpression lines (B76) were significantly less than those of the control (A8) from 1 to 5 h in all three lines (Fig. 1f).

### SAPK10 is Expressed in Rice Guard Cells and SAPK10 Overexpression Lines Present Lower Water Loss in Detached Leaves

**Fig. 2:**
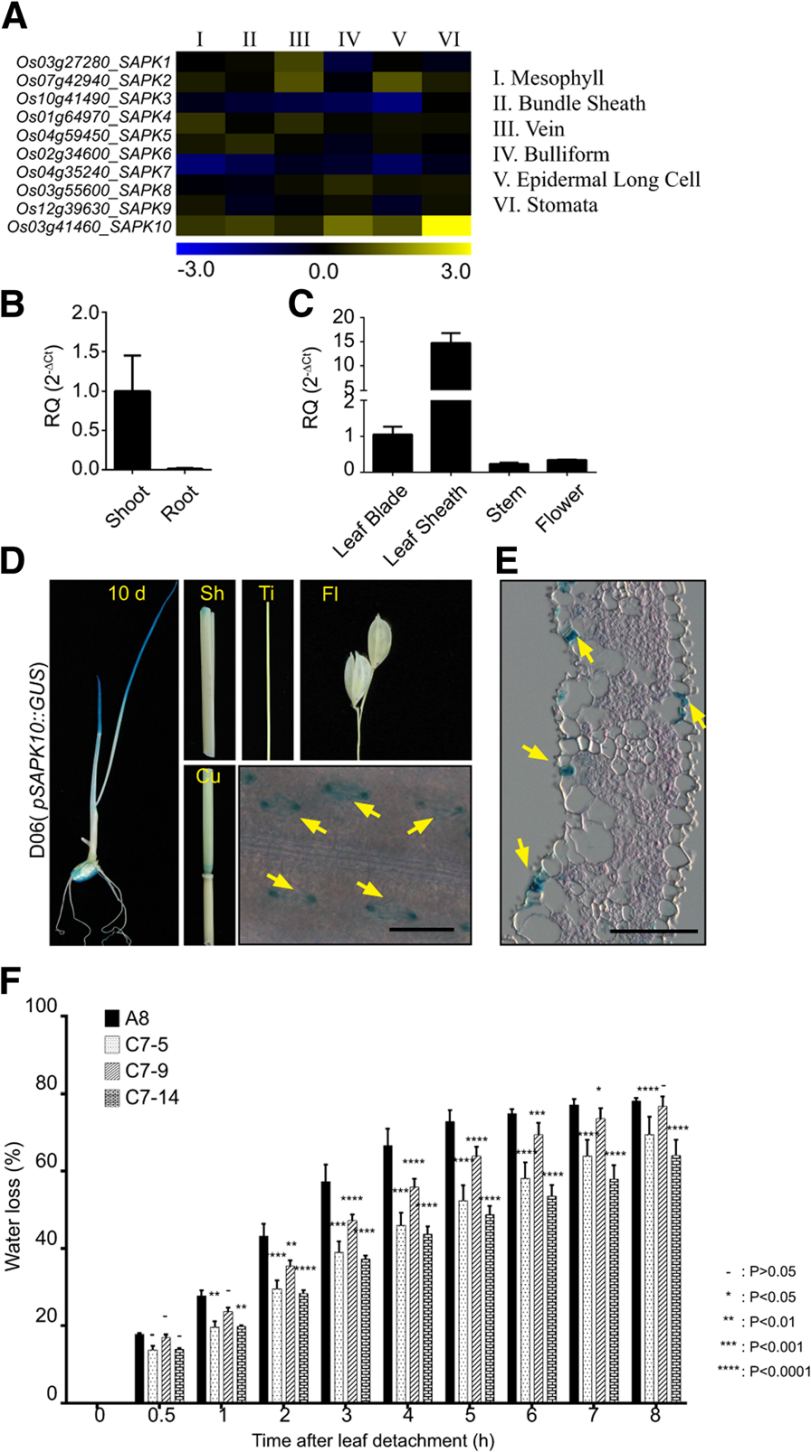
*SAPK10* is expressed dominantly in rice guard cells and *OsSAPK10*-overexpressing transgenic rice presents less water loss. **a** Gene expression profiles of ten SAPKs in different rice cell types were presented as a heat map by using transcriptome analysis data from rice cells, black to yellow displayed increasing, black to blue displayed decreasing. **b** RT-qPCR analysis of *OsSLAC1* gene expression in two-week-old seedlings. The generated RQ (relative quantity, 2^-ΔCt^) was compared to the value of expression on shoot. *OsUbiqutin5* was used as endogenous control. The values depict the average and ± SD with 3 replications. **c** RT-pPCR analysis of OsSLAC1 gene expression in different tissues of mature rice (80 days old). The values were compared to the expression level on leaf blade. **d** Histochemical β-glucuronidase analysis of *SAPK10* promoter-GUS transgenic rice. Left image is a 10-day-old seedling. Sh (sheath), Ti (tiller), Fl (flower), Cu (culm) and leaf blade epidermis. Scale bars are 50 μm. **e** A section of paraffin-embedded leaf blade stained with X-Gluc and eocin. Yellow arrows indicate guard cells. Scale bars are 50 μm. **f** Water loss assays of *SAPK10*-overexpressing transgenic lines (C7–5, 9 and 14) and control line (A8: empty vector transgenic rice). The picks depict the average and ± SD with 3 replications. ANOVA analysis was performed by comparing with water loss of A8 plant

OST1 (SnRK2–6) is a crucial positive regulator of ABA signal transduction in Arabidopsis guard cells (Brandt et al. 2012; Geiger et al. 2009; Mustilli et al. 2002). Rice orthologous of Arabidopsis SnRK2s are called Osmotic Stress/ABA-Activated Protein Kinases (SAPKs) and ten *SAPK*s are present in the rice genome (Kobayashi et al. 2004). We analyzed previously published rice cell-specific microarray data (GEO accession: Go Series GSE13161) to determine which of the ten *SAPK*s are expressed in rice guard cells (Jiao et al. 2009). We found that, of the ten *SAPKs*, *SAPK10* was most prominently expressed in rice guard cells (Fig. 2a). Thus, we experimentally confirmed the expression of *SAPK10* in different tissues using RT-qPCR. *SAPK10* expression was predominant in shoots compared to roots of young rice seedlings (two weeks old) (Fig. 2b). *SAPK10* expression levels were higher in leaf tissues (including leaf blade and sheath) than in other tissues of mature rice (80 days old) (Fig. 2c). Results were similar to microarray data base search at Genevestigator (Additional file 6: Figure S6C). To experimentally validate the guard cell-specific expression of *SAPK10*, we generated transgenic rice lines expressing *GUS* driven by the *SAPK10* promoter (*pOsSAPK10::GUS*, named D06). The *SAPK10* promoter drove expression of *GUS* in the seeds and shoots of young seedlings and in the leaf blade and node of mature plants predominantly, even though lower levels of expression were also observed in the sheath and culm of the mature plant (80 days old) (Fig. 2d). However, we could not observe the expression of SAPK10 in flower and tiller. Microscopic observation of the leaf epidermis and leaf blade sections showed that X-Gluc staining was detected in guard cells predominantly (Fig. 2e). We generated transgenic rice lines overexpressing *SAPK10* and three *SAPK10* over-expression lines named C7 were selected for water loss assays. The expression levels of SAPK10 were confirmed by RT-qPCR analysis (Additional file 4: Figure S4B). All three *SAPK10*-overexpression lines lost significantly less water than the control (A8) (Fig. 2f).

### SAPK10 Interacts With and Phosphorylates OsSLAC1

**Fig. 3:**
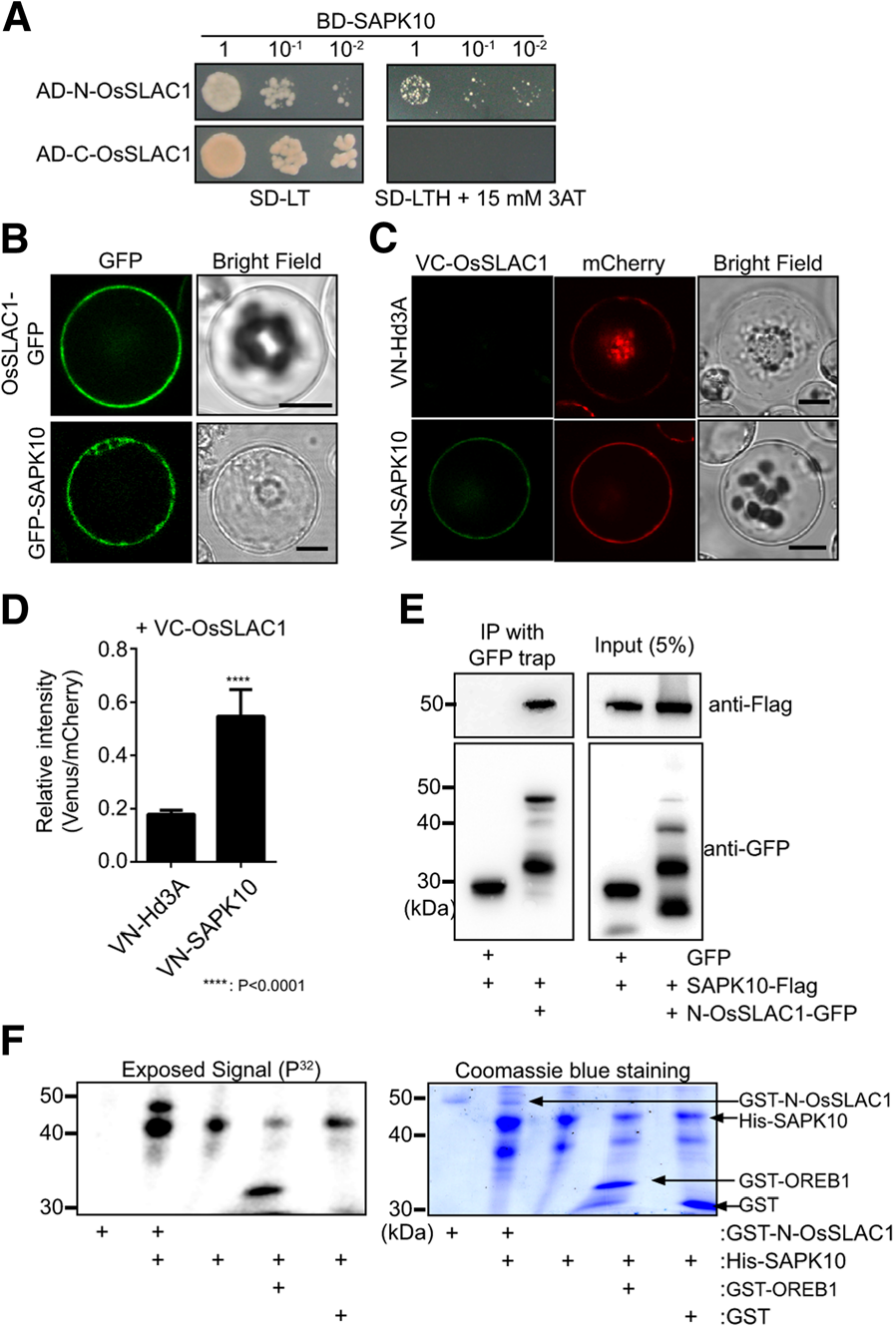
SAPK10 physically interacts with OsSLAC1 and phosphorylates the N-terminal region of OsSLAC1 in vitro. **a** Yeast two hybridization assays. SAPK10 was used as bait (BD-SAPK), and the N-terminal region and C-terminal regions of OsSLAC1 were used as prey (AD-N-OsSLAC2 and AD-C-OsSLAC1). **b** Subcellular localization of SAPK10-GFP in rice protoplasts. **c** BiFC analyses of VN-SAPK10 and OsSLAC1-VC using rice protoplasts. ER-mCherry was used as an internal control. VN-Hd3A was used as a negative control. Scale bars are 10 μm. **d** The relative BiFC signal intensities depict the average ± SD of over 20 cells emitting the fluorescence signals. The value was calculated as Venus signal/mCherry signal. **e** Co-IP analysis using GFP-N-terminal SLAC1 and SAPK10-Flag coexpressed in rice protoplasts. These experiments were repeated three times and a representative result was presented. **f** In vitro kinase assay for His-SAPK10 activity with GST-N-OsSLAC1. The ^32^P radioactivity was detected using a phosphor image analyzer (upper panel) and polyacrylamide gel was stained using Coomassie brilliant blue R250 (lower panel). GST-N-terminal region OREB1 (GST-N-OREB1) was used as a control

When OST1 is activated by ABA signaling, it interacts with and phosphorylates AtSLAC1, thereby activating AtSLAC1 and inducing stomatal closure. Thus, we examined whether SAPK10 can interact with and phosphorylate OsSLAC1. First, we performed yeast two-hybridization assays. Since OsSLAC1 is a plasma membrane protein, the N-terminal and C-terminal regions (N-OsSLAC1 and C-OsSLAC1) where OsSLAC1 protrudes into cytosol were cloned and used as prey. Only N-OsSLAC1 demonstrated a weak interaction with SAPK10 (Fig. 3a).

We further investigated the interaction and subcellular localization of this complex using a bimolecular fluorescence complementation (BiFC) assay. A yellow fluorescence signal was observed on the plasma membrane when VC-OsSLAC1 and VN-SAPK10 were co-expressed in rice protoplasts but not in the negative control (VN-Hd3A and VC-OsSLAC1). The fluorescence values of VN-SAPK10 and VC-SLAC1 were three times higher than VN-Hd3A and VC-SLAC1 (Fig. 3c and d). We also characterized the subcellular localization of GFP-SAPK10, which was detected in the cytosol of rice protoplasts (Fig. 3b). Co-immunoprecipitation (co-IP) analysis also confirmed the physical interaction between N-OsSLAC1-GFP and SAPK10-Flag in rice protoplast (Fig. 3e).

To explore whether SAPK10 is able to phosphorylate OsSLAC1, we expressed glutathione S-transferase (GST)-tagged N-OsSLAC1 (GST-N-OsSLAC1) and 6XHis-tagged SAPK10 (His-SAPK10) in *E. coli* and used the purified proteins to carry out an in vitro phosphorylation assay. His-SAPK10 phosphorylated itself, GST-N-OsSLAC1 and the N-terminal region of OREB1 (GST-N-OREB1), used as positive control (Fig. 3f) (Chae et al. 2007). Based on these results, we conclude that SAPK10 is a functional orthologue of OST1, which physically interacts with OsSLAC1 and can phosphorylate its N-terminal region.

### OsPP2C50 and OsPP2C53 are Localized in the Cytosol and are Expressed Predominantly in Guard Cells

**Fig. 4:**
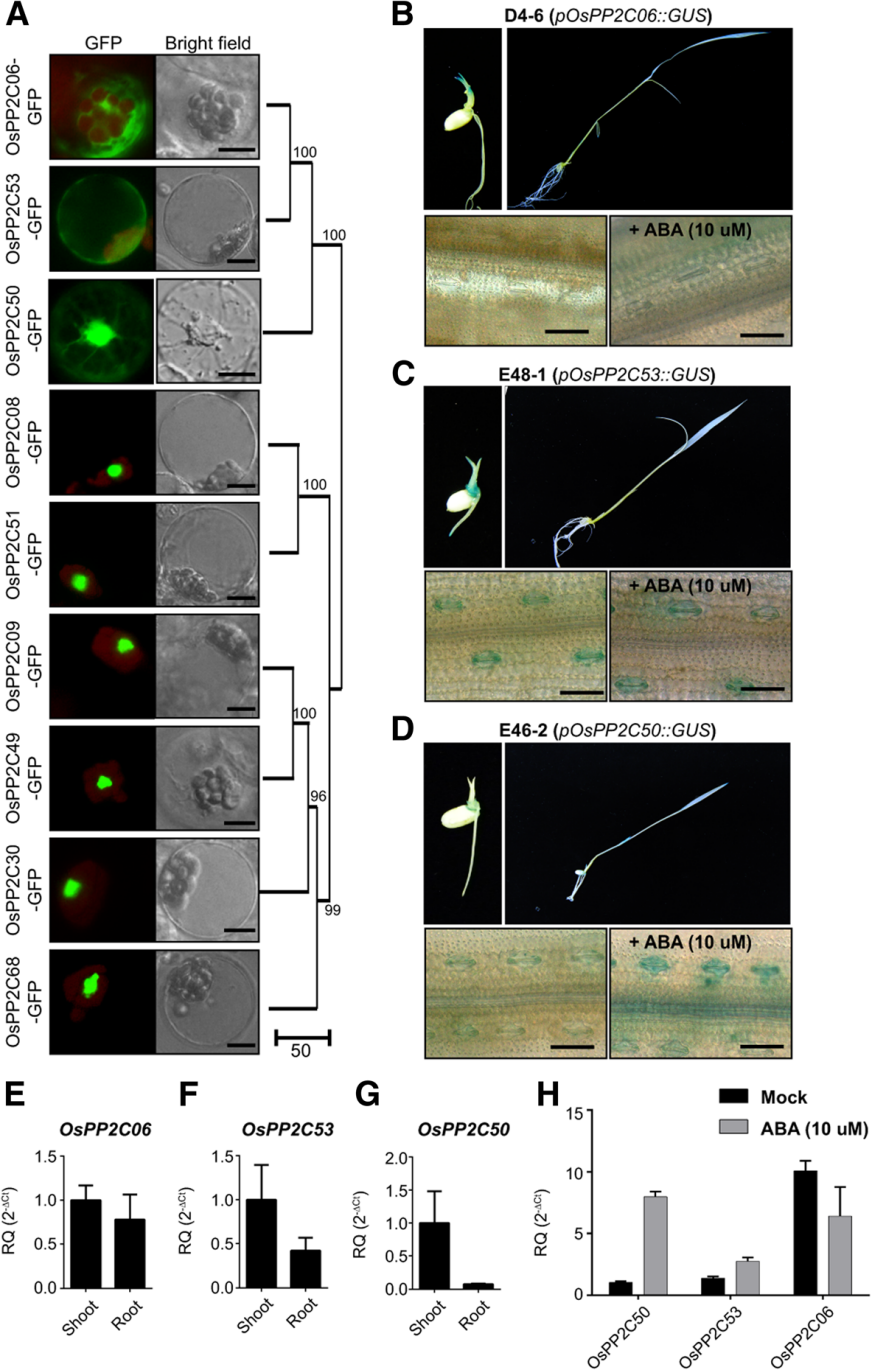
Expression of *OsPP2C50* and *OsPP2C53* in rice guard cells and subcellular localizations of OsPP2CAs. **a** Subcellular localizations of nine OsPP2CAs were observed in rice protoplasts. Scale Bars = 10 μm. **a** Subcellular localization of OsPP2CA-GFPs in rice protoplasts. **b**, **c**, **d** Histochemical β-glucuronidase analysis of *OsPP2CA* promoter-GUS transgenic rice. Leaf surface of transgenic rice harboring *OsPP2C06 promoter-GUS* (D4–6), *OsPP2C53 promoter-GUS* (E48–1), and *OsPP2C50 promoter-GUS* (E46–2) with or without ABA treatments were stained with X-Gluc. Scale bars are 50 μm. **e**, **f**, **g** RT-qPCR analysis of *OsPP2C06, OsPP2C50* and *OsPP2C53* using two-week-old seedlings. The generated RQ (relative quantity, 2^-ΔCt^) was compared to the value of expression on shoot. *OsUbi5* was used as endogenous control. The values depict the average and ± SD with 3 replications. **h** RT-qPCR analysis of *OsPP2C06, OsPP2C50* and *OsPP2C53* using two-week-old seedlings treated or not treated with ABA. The detected RQ (relative quantity, 2^-ΔCt^) displayed *OsUbi5* was used as endogenous control. The values depict average and ± SD with 3 replications

Clade A phosphatase 2Cs (PP2CAs) are negative regulators of SAPK/SnRK2 in ABA signaling, in both Arabidopsis and rice, and we identified nine OsPP2CAs in the rice genome (Kim et al. 2012). To identify which OsPP2CAs among 9 OsPP2CAs function in guard cell to regulate SAPK10 and OsSLAC1, we surveyed the subcellular localization of OsPP2CAs and the expression of the *OsPP2CA*s in guard cells. First, we characterized the subcellular localization of all nine OsPP2CAs in rice protoplasts. The OsPP2CAs were clearly divided into two groups based on subcellular localization: one with nucleus-specific localization, and the other with non-nucleus-specific localization, resembling cytosolic, ER-like or Plasma membrane localization and distributed ubiquitously in cell (Fig. 4a). These two groups corresponded to the two clades observed in phylogenetic analysis which are presented in the right side of subcellular localization (Fig. 4a). As candidates to regulate SAPK10, we chose the three OsPP2CAs (OsPP2C06 (Os01g40094), OsPP2C53 (Os05g51510) and OsPP2C50 (Os05g46040)) not located specifically in the nucleus, and then explored whether they were expressed in rice guard cells. We generated transgenic rice lines named D04, E48, E46 harboring *pOsPP2C06::GUS*, *pOsPP2C53::GUS* and *pOsPP2C50::GUS* reporter vectors, respectively. We found that the promoters of *OsPP2C50* and *OsPP2C53* drove expression of *GUS* in guard cells, but that of *OsPP2C06* did not, regardless of ABA treatment (Fig. 4b, c, d). The promoter of *OsPP2C50* induced the expression of *GUS* in rice guard cells in an ABA-dependent manner (Fig. 4d), whereas *OsPP2C53* constitutively drove expression of *GUS* in rice guard cells regardless of ABA treatment (Fig. 4c). RT-qPCR analysis confirmed that *OsPP2C50* is predominantly expressed in shoots, whereas *OsPP2C06* and *OsPP2C53* are expressed in both shoots and roots (Fig. 4e, f, g). Expression of *OsPP2C06* was not induced by ABA treatment. Without ABA treatment condition OsPP2C53 was expressed 30% more than OsPP2C50, but in ABA treatment condition, *OsPP2C50* was induced much strongly than that of *OsPP2C53* (Fig. 4h).

### OsPP2C50 and OsPP2C53 Physically Interact with SAPK10 and their Overexpression Lines Present Higher Water Loss in Detached Leaves and Drought Sensitivity

**Fig. 5:**
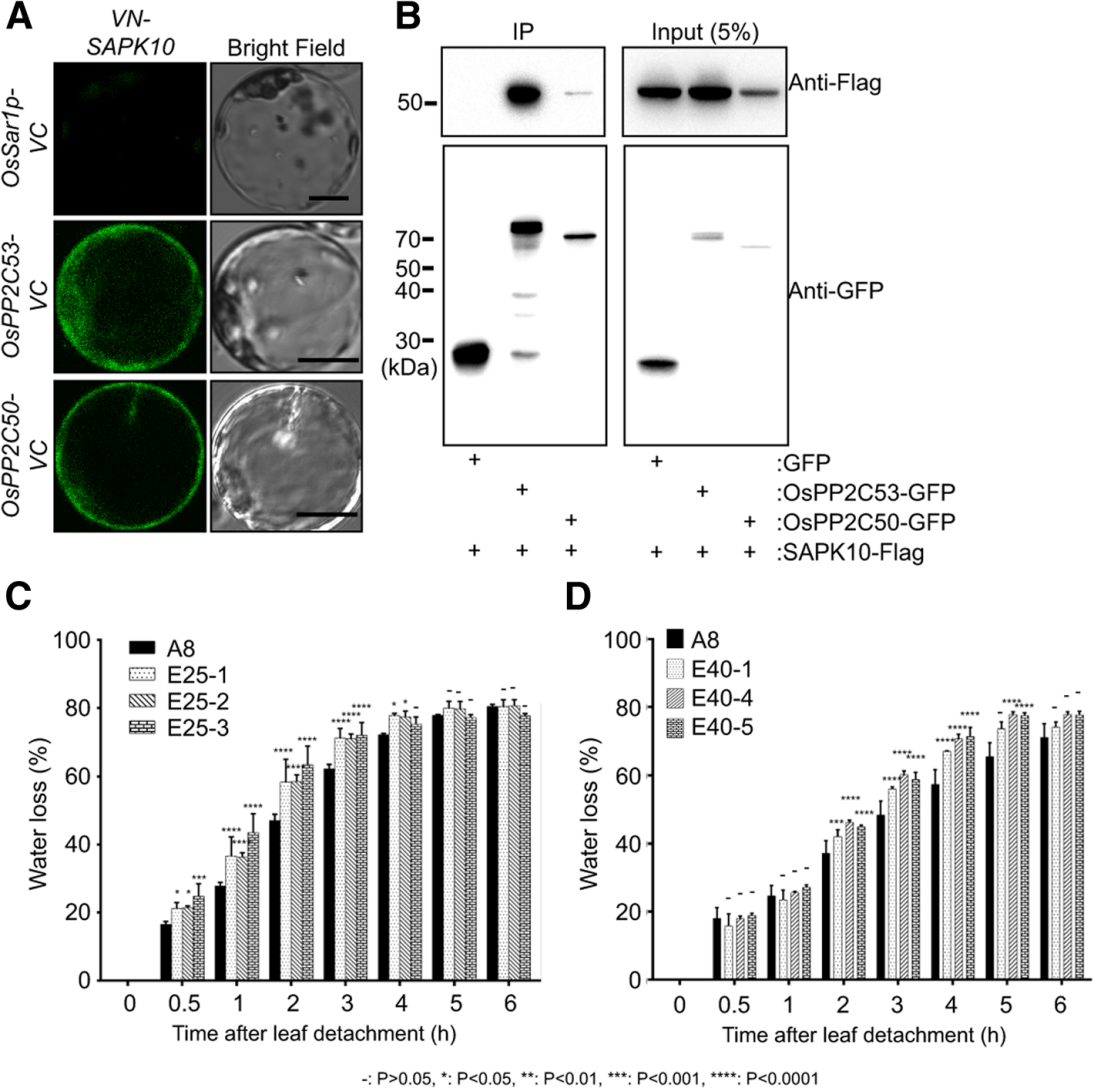
OsPP2C50 and OsPP2C53 physically interact with SAPK10 and their overexpression promotes water loss. **a** BiFC analysis of VN-SAPK10 and OsPP2C53-VC or OsPP2C50-VC. OsSar1p-VC was used as a negative control. Scale bars are 10 μm. **b** Co-IP analysis using OsPP2C53-GFP or OsPP2C50-GFP and SAPK10-Flag expressed in rice protoplasts. These experiments were repeated three times and a representative result was presented. **c** Water loss assays of *OsPP2C53*-overexpressing transgenic lines (E25–1, 2 and 3) and control line (A8: empty vector transgenic plant). **d** Water loss assay of *OsPP2C50*-overexpressing transgenic lines (E40–1, 4 and 5) and control line (A8: empty vector transgenic plant). The values depict the average and ± SD with 3 replications. ANOVA analyses were performed by comparing with water loss of A8 plant.

PP2CAs can physically interact with and negatively regulate SnRK2/SAPK activity in Arabidopsis and rice (Bhatnagar et al. 2017). Thus, we investigated whether OsPP2C50 and OsPP2C53 can physically interact with SAPK10. First, we performed yeast two-hybridization using SAPK10 as bait (BD-SAPK10) and nine OsPP2CAs as prey (AD-OsPP2CA). OsPP2C6, 50, 53 and 09 showed colony growth on selection media, indicating that they interacted with SAPK10, but other OsPP2CAs did not (Additional file 2: Figure S2). BiFC assays also revealed fluorescent signals between VN-SAPK10 and OsPP2C53-VC or OsPP2C50-VC in rice protoplasts, but not between VN-SAPK10 and Sar1p-VC, which was used as negative control. The VN-SAPK10-OsPP2C53-VC and VN-SAPK10-OsPP2C50-VC complexes were localized in the cytosol of rice protoplasts (Fig. 5a). For further interaction studies, we performed co-IP assays. SAPK10-Flag was detected when OsPP2C53-GFP or OsPP2C50-GFP were precipitated with an anti-GFP trap (Fig. 5b). Furthermore, it was confirmed that OsPP2C50 and OsPP2C53 could inhibit the phosphorylation function of SAPK10 in in vitro phosphorylation assay (Additional file 5: Figure S5). Taken together, *OsPP2C53* and *OsPP2C50* are expressed in rice guard cells and can interact with *SAPK10* as negative regulators.

We also generated transgenic rice lines E25 and E40 overexpressing *OsPP2C53* and *OsPP2C50*, respectively. Three over-expression lines were selected for water loss assays based on their gene expression levels (Additional file 4: Figure S4C and D). Water loss of the E25 and E40 lines was significantly higher than that of the control in all lines tested (Fig. 5c, d). In addition, the transgenic lines showed drought hypersensitivity at the young seedling stage (Additional file 3: Figure S3). Taken together, OsPP2C50 and OsPP2C53 might negatively regulate SAPK10 and OsSLAC1 in stomatal closure.

### OsPP2C50 and OsPP2C53 Directly Interact with the N-Terminal Region of OsSLAC1

In Arabidopsis, AtPP2CA interacts with SLAC1 during ABA signaling, and ABI1 can directly dephosphorylate the N-terminal region of SLAC1 even in response to Ca^2+^ signaling (Brandt et al. 2012; Geiger et al. 2009; Lee et al. 2009; Maierhofer et al. 2014).

**Fig. 6:**
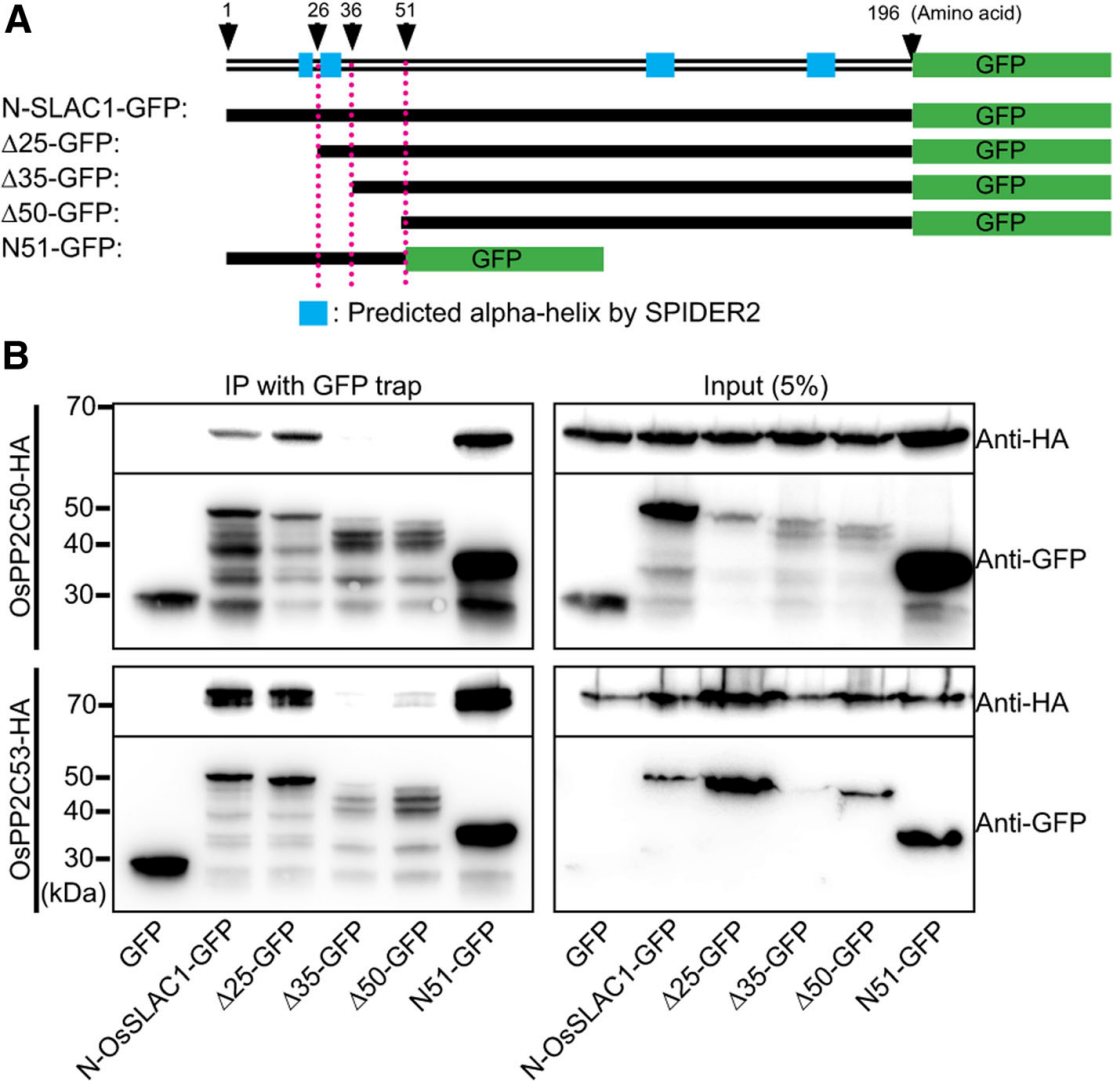
OsPP2C50 and OsPP2C53 directly interact with OsSLAC1-N-terminal region. **a** Diagram of deletion mutant constructs of OsSLAC1 used for co-IP. **b** Co-IP analysis using OsPP2C50-HA and several deletion mutant forms of N-SLAC1-GFP co-expressed in rice protoplasts. These experiments were repeated three times and a representative result was presented

Accordingly, we explored the physical interaction between OsPP2C50 and OsPP2C53 using Co-IP analysis. First, we identified that N-OsSLAC1-GFP can physically interact with OsPP2C50-HA and OsPP2C53-HA. Next, we identified the interaction motif of N-OsSLAC1 responsible for binding to OsPP2C50-HA and OsPP2C53-HA. OsPP2C50-HA and OsPP2C53-HA co-precipitated with N-OsSLAC1-GFP, the N-terminal 25 amino acid deletion of OsSLAC1 (Δ25), and N51-GFP, containing only the first 51 amino acids of OsSLAC1 (Fig. 6a). However, neither the Δ35 nor Δ50 N-terminal deletions were co-precipitated. These results revealed that the N-terminal 26–35 amino acids of OsSLAC1, which are predicted to form the second alpha-helix in the N-terminal region of OsSLAC1 (Fig. 6b), play an important role in the interaction with OsPP2C50 and OsPP2C53.

## Discussion

More than 95% of water is evaporated through stomatal pores in plants. Stomatal movement therefore has crucial functions in the water usage efficiency of plants (Schroeder et al. 2001). Thus far, the molecular mechanisms for stomatal closure and opening have been most studied in the model plant Arabidopsis, whereas studies in monocot plants, including most crops such as rice, maize, wheat and barley, have been limited (Chen et al. 2017; Hwang et al. 2013; Moon et al. 2017; Raissig et al. 2017; Sun et al. 2016). In this study, we unraveled the core ABA signaling components and networks functioning in stomatal closure of rice, a model monocot plant. Our systematic and comparative functional analysis suggests that rice and Arabidopsis have evolutionally conserved ABA signaling components, which interact in similar interaction networks, for stomatal closure, despite their stomatal complexes having different anatomical structures.

### ABA Signaling Components Functioning in Stomatal Closure are Evolutionally Conserved Between Monocots and Dicots

Earlier studies have identified SLAC1 homologs in Arabidopsis, rice, and barley based on their amino acid similarity and genetic analysis (Negi et al. 2008; Saji et al. 2008; Vahisalu et al. 2008). OsSLAC1 is the most similar to Arabidopsis SLAC1 of the rice OsSLAHs (Vahisalu et al. 2008). We additionally demonstrated that *OsSLAC1* was specifically expressed in rice guard cells, in contrast to the other *OsSLAHs*. Even though the SLAC1 proteins of monocot and dicot plants have highly conserved amino acid sequences, they have different electrophysiological characteristics. Monocot SLAC1 requires extracellular nitrate to activate it under ABA whereas SLAC1 from dicots does not (Hedrich and Geiger 2017; Schafer et al. 2018). Also monocots and dicots have different anatomical structure in stomatal complex which monocots consists of a pair of dumbbell-shaped guard cells and a pair of subsidiary cells, in contrast to the kidney-shaped guard cells typical of dicots. Accordingly, we thought that the ABA signaling components, pathways and molecular mechanisms functioning in the guard cells of monocots and dicots might differ.

Here, we identified signaling networks consisting of SAPK10, OsPP2C50 and OsPP2C53 as the core ABA signaling components related to the regulation of OsSLAC1 in rice guard cells. We demonstrated that these genes correspond to Arabidopsis *OST1*, *ABI1* and *ABI2*, based on phylogenetic analysis, guard cell-specific gene expression, subcellular localization, and physical interaction and water loss assays of transgenic plants overexpressing those genes. Taken together, the core ABA signaling components functioning in guard cells are highly conserved in terms of gene functions, expression profiles, interaction and structures in monocots and dicots. Thus, the molecular mechanisms of ABA signaling in guard cells might be conserved among monocots and dicots, although further studies regarding these components might discern differences in the mechanistic details of the two.

### Clade A PP2Cs Localized Ubiquitously in Cells Might Play Roles in Diverse Signaling Pathways

In our results nine OsPP2CAs can be divided into two groups based on their subcellular localization patterns. One is confined in nuclei and the other is localized ubiquitously in the cell, including nuclei, cytosol and ER. Similar subcellular localization patterns have also been observed in Arabidopsis (Umezawa et al. 2009). Thus, the subcellular localization patterns of PP2CAs might be functionally important and evolutionary conserved in rice and Arabidopsis. Proteins that interact with PP2CAs and signaling molecules are differentially distributed in the subcellular organelles. For example, OsPP2C51 which is localized specifically in nuclei can interact with nuclear localized transcription factor OsbZIP 10 (Bhatnagar et al. 2017). ABI1, known to be localized in the cytosol, can interact with the N-terminal region of AtSLAC1 that protrudes into the cytosol in Arabidopsis (Brandt et al. 2012; Lee et al. 2009). It was known that stomatal closure and opening are affected by diverse environmental cues such as ABA, Ca^2+^, CO2 and H_2_O_2_ and by signaling components such as SnRK2, CIPK, CDPK, and MAPKs. OsPP2C50 and OsPP2C53 among OsPP2CAs might be localized ubiquitously in cell such as the cytosol, ER, nuclei or plasma membrane. Thus they might interact with diverse signaling components and play a role as common negative regulators of stomatal opening in different signaling. That is one of the reasons why plant overexpressing them showed significant drought sensitivity and water loss phenotype. It also show that OsPP2C50 and OsPP2C53 are common negative regulator of stomata closure signaling that the interaction domain of N-terminal OsSLAC1 is close to amino acid S59 which is similar position S50 of AtSLAC1 phosphorylated by CDPKs. Two OsPP2Cs interacted at similar position of N-terminal OsSLAC1 (25–36) could inhibit the phosphorylation function of SAPK10, which could phosphorylate the N75-OsSLAC1 (Additional file 5: Figure S5).

Differences between *OsPP2C50* and *OsPP2C53* in terms of ABA-dependent gene expression suggest they might have different functions in ABA signaling. Without ABA condition both OsPP2C50 and OsPP2C53 might function as suppression components in stomata closure and under the high ABA condition the ABA-dependently expressed OsPP2C50 might play a role as a major negative inhibitor in guard cell closure.

One of the biggest challenges facing agricultural researchers is to improve water usage efficiency of crops because the cereal supply for humans is threatened by water deficiency caused by global warming. Studies on stomatal movement regulation are a major research target for improving water use efficiency because stomatal pores are nearly the only gate by which water leaves the plant. In this study, we identified two PP2CAs, OsPP2C50 and OsPP2C53 as very strong negative regulators of stomatal closure functioning in rice guard cells. Thus, these two genes might be promising targets for engineering improved water use efficiency in monocots.

## Conclusion

In this study we found that OsPP2C50 and OsPP2C53 specifically expressed in rice guard cell and is major negative regulators of ABA signaling regarding stomata closing in rice. OsPP2C50 and 53 can interact with the N-terminal of OsSLAC1 directly or can interact with SAPK10 which is specifically expressed in rice guard cell.

## Materials and Methods

### Plant Growth Conditions and Generation of Transgenic Plants

Husked rice seeds (*Oryza sativa subsp. japonica* cv. Dongjin) were sterilized by immersion in 70% ethanol for 30 s, and in 2% NaClO for 40 min. After washing 5 times with distilled water, the sterilized seeds were planted on half-strength (½) Murashige and Skoog (MS; Duchefa Biochemie, Netherlands) medium supplemented with 0.4% phytagel and adjusted to pH 5.8. The plants were grown in a culture room at 28 °C under 16/8 h light/dark cycles.

1000~1500 bp upstream regions of each of *OsSLAC1*, *SAPK10*, *OsPP2C06*, *OsPP2C53*, and *OsPP2C50* were isolated from rice genomic DNA by polymerase chain reaction (PCR) amplification with the primers indicated in Additional file 8: Table S1 to generate the transgenic plants harboring promoter-GUS reporters. The PCR products were cloned using the pENTR/D-Topo cloning kit (Invitrogen, USA), and transferred to the promoter region of the pBGWSF7 vector, a GUS-expressing binary vector, via an LR recombination reaction using the Gateway system (Invitrogen, USA) (Karimi et al. 2002). The full-length cDNAs were cloned using the pENTR/D-Topo cloning kit and then transferred into the pGA2897 vector harboring the Maize UBIQUITIN promoter (Kim et al. 2012). The resulting plasmids were transferred into *Agrobacterium* competent cells (*Agrobacterium tumefaciens* strain LBA4404). Rice transgenic plants were generated using the *Agrobacterium*-mediated co-cultivation method (Han et al. 2012; Toki et al. 2006). Transformants were screened based on phosphinothricin (PPT) resistance and hygromycin resistance for pBGWFS7 and pGA2897, respectively.

### Total RNA Isolation and Reverse Transcription Quantitative PCR

Total RNA was isolated from five-day-old rice seedlings for over-expression line selection and from two-week-old rice seedlings and mature plants for identifying expression levels using the Qiagen RNeasy Plant Mini Kit (QIAGEN, USA). Contaminating genomic DNA was digested with Recombinant DNase 1 (RNase-free) (TAKARA BIO, Japan). From total RNA (3 μg), first strand cDNA was synthesized with oligo dT and random hexamers using the SuperScript**™** Reverse Transcriptase first strand synthesis system (Invitrogen, USA) following the manual. Reverse transcription quantitative PCR (RT-qPCR) assays using the iQ**™** SYBR® Green Supermix (Biorad, USA) were employed in this study.

The reaction mixture was subjected to denaturation at 95 °C for 3 min, followed by 30 ~ 40 cycles of denaturation at 94 °C for 10 s, annealing at 58 °C for 15 s, and elongation at 72 °C for 20 s. Triplicate quantitative assays were performed on each cDNA sample. Sequences of the primers used are listed in Additional file 8: Table S1. RT-qPCR was performed with internal primers, *UBI5* was used as the control, and relative gene expression was analyzed by the delta-delta Ct method (Dooms et al. 2014).

### Rice Protoplast isolation and PEG-Mediated Transient Transformation

To isolate rice protoplasts, plants were germinated and grown for 10 days on ½ MS medium supplemented with 0.4% phytagel adjusted to pH 5.8 and then subjected to 8 days in the dark followed by 2 days under a 16/8 h light/dark cycle at 28 °C in an incubation room. The resulting plants were chopped into approximately 1 mm pieces and immediately transferred into freshly prepared enzyme solution (1.5% cellulose R-10, 0.75% Macerozyme R-10, 0.6 M mannitol, 10 mM CaCl_2_, 0.1% bovine serum albumin (BSA), 0.06% β-mercaptoethanol and 10 mM 2-(N-morpholino) ethanesulfonic acid at pH 5.7). After gentle shaking at 60–80 rpm for 4 h at room temperature, protoplasts were isolated by passing through a 100 μm diameter mesh and sedimented by centrifugation at 100×*g*. After washing with W5 solution (154 mM NaCl, 125 mM CaCl_2_, 5 mM KCl, and 2 mM MES adjusted to pH 5.7), isolated protoplasts were resuspended in MaMg solution (0.6 M mannitol, 15 mM MgCl_2,_ and 5 mM MES at pH 5.7) at a concentration of 3–5 × 10^6^ cells mL^− 1^. Then, 0.3 mL of suspended cells was gently mixed together with the DNA plasmids indicated and freshly prepared 0.33 mL polyethylene glycol electrolyte (PEG) solution (40% (*w/v*) polyethylene glycol (MW 6000), 0.1 M CaNO_3_, and 0.4 M mannitol). After 25 min incubation at room temperature, the samples were serially diluted with W5 solution (630 mL, 1200 mL and 2500 mL) and mixed for 10 min each time. Finally, the protoplasts were incubated with W5 solution at 28 °C until use (Kim et al. 2015).

### Subcellular Localization and BiFC

To observe subcellular localization of GFP-fused proteins, the coding sequences (CDS) of selected genes were inserted into pENTR/D-topo vectors (Invitrogen, CA, USA). CDSs were transferred into pMDC43 and pMDC83 vectors (Curtis and Grossniklaus 2003) via LR recombination reactions using the Gateway system (Invitrogen, CA, USA) (Curtis and Grossniklaus 2003). After purification, the constructs were introduced into rice protoplasts using a PEG-mediated method. GFP and mCherry signals were captured using a Leica TCS SP8 laser scanning confocal microscope and fluorescence microscopy (Carl Zeiss, DE/Axio imager M1). The combinations of excitation wavelength / detection range of emission on the confocal microscopy were 488 nm (solid state laser)/ 493 to 530 bandpass for GFP and 552 nm (solid state laser)/ 603 to 662 bandpass for mCherry. For fluorescence microscopy, filter sets were XF116 (exciter, 474AF20; dichroic, 500DRLP; emitter, 510AF23) for GFP and XF33/E (exciter, 535DF35; dichroic, 570DRLP; emitter, 605DF50 [Omega]) for mCherry. The detected images are presented in pseudocolor. To quantify the rate of co-localization between two different proteins on an image, images were analyzed by the Leica Application SuitX analysis program with default settings (threshold 30%, background 20%).

To observe interactions between proteins using bimolecular fluorescence complementation (BiFC), inserts were introduced into pENTR-D-TOPO vectors (Invitrogen, CA) and then transferred to their destination vectors (pGEM-gw-VC or pGEM-VC-gw vectors for Venus Carboxyl-terminus (VC) tagging, and pGEM-VN-gw or pGEM-gw-VN vectors for Venus Amino-terminus (VN) tagging using LR recombination (Promega, WI, USA). All BiFC gateway vectors (pGEM-gw-VN, pGEM-VN-gw, pGEM-gw-VC, and pGEM-VC-gw) were prepared to amplify Gateway cassette regions (35S promoter to Nos-terminator) of pDEST-VYNE, pDEST-VYNE (R), pDEST-VYCE, and pDEST-VYCE(R) by PCR reaction with primers (5′-*Hind*III-35S and 3′-*Eco*RI-nos) and ligate with the pGEM-T Easy vector (Promega, WI, USA) (Gehl et al. 2009). Fluorescence signals were captured using a Leica TCS SP8 laser scanning confocal microscope. The combination of excitation wavelength / detection range of emission for Venus signals was 488 nm (solid state laser)/ 505 to 561 bandpass. Signal intensities on captured images were analyzed using Leica Application SuiteX.

### In Vitro Phosphorylation Assay

In vitro phosphorylation assays of SAPK10 activity on OsSLAC1 were carried out as previously described (Bhatnagar et al. 2017; Chae et al. 2007). The CDSs of SAPK10 and the N-terminal region of OsSLAC1 were cloned into pRST-A and pGEX-5x-1, respectively. 6xHis-tagged SAPK10 and GST-tagged proteins (N-OsSLAC1 and N-OREB1) were purified using Ni-NTA resins (Thermo Scientific, IL, USA) and Glutathione Sepharose High Performance resins (GE Healthcare, Sweden), respectively. Aliquots of His-fused SAPK10 kinase and substrates were incubated in phosphorylation buffer (10 μCi γ-32P-ATP, 10 μM ATP, 20 mM Tris–HCl pH 7.0, 5 mM MgCl_2_ or MnCl_2_) for 30 min at 30 °C. After stopping the reaction by addition of 10 mM EDTA, the products were analyzed by sodium dodecyl sulfate polyacrylamide gel electrophoresis (SDS-PAGE). Gels were stained with Coomassie Brilliant Blue R-250, dried, and analyzed with a phosphoimage analyzer (Personal Molecular Imager FX system, Bio-Rad, Benicia, CA, USA).

### Histochemical GUS Expression Analysis

To test gene expression, seedlings or mature GUS-expressing transgenic plants were stained with 5-Bromo-4-chloro-3-indolyl-β-D-glucuronide (X-Glu), a substrate of GUS. Plant materials were briefly washed with 90% acetone, then analyzed following the methods previously reported using GUS-staining buffer [2 mM X-Gluc, 50 mM NaSO_4_, 0.1% Triton X-100, 0.5 mM K_3_Fe^III^(CN)_6_, and 0.5 mM K_3_Fe^II^(CN)_6,_ adjusted to pH 7.0] (Kim et al. 2015). GUS-stained plants and tissues were fixed by washing several times with 70% ethanol until the chlorophyll was completely removed from the tissue. Paraffin sectioning and eosin staining were completed by conventional methods. In brief, samples were embedded in paraffin and 4 μm cross-sections were made using a RM2255 microtome (Leica, Germany). These slices were stained with eosin following standard manual, mounted with Permount (Thermo Fisher Scientific, USA) and observed using a Zeiss AxioCam MRc CCD camera (Carl Zeiss, Jena, Germany).

### Co-Immunoprecipitation Assay

To perform immunoprecipitation experiments, we used GFP-trap (Chromotek, Germany) for the immunoprecipitation of GFP-fusion proteins. All tested genes were inserted into pENTR/D-topo vectors (Invitrogen, CA, USA) and then recombined with pGEM-gw-GFP or pGEM-GFP-gw vectors for GFP fusion, pGEM-gw-3xHA vector for HA tagging, and pGEM-gw-Flag vector for Flag tagging using LR recombinase (Invitrogen, CA, USA). To generate pGEM-gw-GFP or pGEM-GFP-gw vectors, the Gateway cassette regions (35S promoter to Nos-terminator) of pMDC83 and pMDC43 were amplified by PCR with primers (5-pMDC and 3-pMDC), and the PCR products were inserted into pGEM-T easy vectors (Promega, WI, USA). To generate pGEM-gw-3xHA and pGEM-gw-Flag vectors, the Gateway cassette regions (*Ubi* promoter to *nos* terminator) of pGA2897were amplified by PCR with primers (5-Ubi and 3-nos-t), and then the PCR products were ligated with the pGEM-T easy vector (Promega, WI, USA). The indicated constructs were introduced into rice protoplasts using the PEG-mediated method, and the transformed protoplasts were incubated at 28 °C. Cellular extracts from transformed protoplasts in immunoprecipitation buffer [150 mM NaCl, 50 mM Tris-HCl at pH 7.5, 1 mM EDTA, 2 mM EGTA, 2 mM MgCl_2_, 0.5% NP40, 0.5% Triton X-100, and 1x protease inhibitor cocktail (complete ULTRA tablet, Roche, IN, USA)] were incubated with pre-cleaned GFP-trap beads at 4 °C for 2 h. After washing 5 times with immunoprecipitation buffer, the precipitated proteins, together with GFP-trap, were subjected to SDS-PAGE and immunoblot analysis. Precipitated GFP, HA, or Flag-tagged proteins were detected with anti-GFP rabbit antibody (Life Technologies, OR, USA), anti-HA rat antibody (Roche, IN, USA), or anti-Flag M2 peroxidase antibody (Sigma), respectively.

### Yeast Two-Hybrid Assay

Yeast two-hybridization assays were performed using the Matchmaker™ GAL4 Two-Hybrid System 3 (Clontech, USA) according to the manufacturer’s manual. The lithium acetate method was used to introduce pGADT7 (as the yeast activation domain) and pGBKT7 (as the yeast binding domain) plasmids into yeast strain *Saccharomyces cerevisiae* strain AH109. Yeast were plated on Yeast Minimal Media/Synthetic Defined (SD) media (Clontech, USA) without leucine and tryptophan, then transferred to selection media without leucine, tryptophan and histidine supplemented with 15 mM 3-amino-1, 2, 4-triazole (3-AT) (Sigma, USA). For yeast spotting assays, exponentially grown yeast cells were harvested and adjusted to OD_600_ 0.5 with sterilized water and diluted 1/10 and 1/100. Yeast cells were spotted onto SD medium without leucine and tryptophan and SD medium without leucine, tryptophan and histidine. Growth was assessed 3 days after spotting.

### Water Loss Assay

For water loss of detached leaves, leaves were removed from A8 (pGA2897 empty vector) transgenic plants as a control and *OsSLAC1, SAPK10* and *OsPP2CA50* and *OsPP2CA53* over-expression lines that had been grown for 10 days in a square box (72 mm X 72 mm X 100 mm) with 1/2 MS medium under long day conditions (16 h light and 8 h dark) at 28 °C in a cultivation room. One day before measurements, the box cover was opened, and plants were allowed to adapt to outside conditions. Detached leaves were placed on a laboratory bench and periodically weighed. The experiment was performed each time with three replicate leaves per line.

### Drought Assay

For drought stress tolerance analysis, seeds from *OsPP2CA53* over-expression lines (E25) and *OsPP2CA50* over-expression lines (E40) were grown in white square pots with soil in a growth chamber with sufficient water until the fourth leaf stage. Each sample was tested in three independent experiments. Watering was stopped for 5 days until leaves completely wilted just before dying, and then the plants were re-watered. After recovery of the plants, the survival rates of the plants were measured.

## Additional Files

Additional file 1: **Figure S1.** OsSLAC1 has the highest homology to AtSLAC1 and is expressed predominantly in leaf tissues. (A) Phylogenetic tree depicting S-type anion channels of Arabidopsis and their homologues in rice. This tree was constructed using the neighbor-joining method in Mega 6.0. (B) Tissue-specific expression data for rice S-type anion channels were analyzed using the Rice X pro database. (TIF 3251 kb)

Additional file 2: **Figure S2.** Yeast two hybridization assay between BD-SAPK10 and AD-OsPP2CAs. SD: Synthetic Drop-out medium, L: leucine, T: Tryptophan, H: Histidine, 3AT: 3-amino-1,2,4-triazole. (TIF 1196 kb)

Additional file 3: **Figure S3.** Transgenic rice overexpressing *OsPP2C50* and *OsPP2C53* present drought hypersensitive phenotype. Pictures showing drought hypersensitivity of *OsPP2C53*-overexpressing transgenic lines (E25–1, 2 and 3) compared to control line (DJ, DongJin) (A) and OsPP2C50-overexpressing transgenic lines (E40–1, 4 and 5) compared to control line (C). (B, D) Survival rate after rewatering the drought treated rice of each transgenic line. These data obtained from 3 independent experiment, *N* > 20. The values depict the average and ± SD. (TIF 6522 kb)

Additional file 4: **Figure S4.** Confirmation of gene overexpression in transgenic rice. All overexpressing transgenic plants were confirmed by RT-qPCR analysis with each specific primer. The expression level of *Ubiqutin5* was used by endogenous control. All values were compared to the value of DJ. (A) OsSLAC1, (B) SAPK10, (C) OsPP2C53, and (D) OsPP2C50. (TIF 1919 kb)

Additional file 5: **Figure S5.** Inhibition of kinase activity of SAPK10 by OsPP2C50 or OsPP2C53 In vitro kinase assay were performed with OsPP2C50 or OsPP2C53. Each protein were used indicated quantity (below) and phosphorylated with SAPK10 in reaction buffer (60 mM NaCl, 20 mM Tris-Cl, pH 7.4, 5 mM MgCl_2_, 10 μM ATP, and 10 μCi γ-32P-ATP) at 30 °C for 30 min. (A) Detected phosphor image, (B) Polyacrylamide gel stained with Coomassie brilliant blue R250. (TIF 2460 kb)

Additional file 6: **Figure S6.** The expression of SAPKs, OsPP2Cs and OsSLAC1/OsSLAHs in anatomy microarray data base. (A,B,C) OsSLAC1/OsSLAHs, OsPP2Cs, and SAPKs expression were search in microarray data base (Genevestigator). (D) The experimental ID (ex_ID) and used GEO numbers of DATA sets. (TIF 5518 kb)

Additional file 7: **Supplementary methods.** Purification of GST-N75-OsSLAC1. To obtain the GST-N75-OsSLAC1, we constructed *pGEX-5x-N75-OsSLAC1*. N-terminal region (N75) of OsSLAC1 was amplified by PCR reaction with specific primers (Additional file 8: Table S1) and harbored in pGEX-5x-1 vector with digestion by restriction enzymes (BamH1 and EcoR1) and ligation. Expressed GST-N75-OsSLAC1 in *E. coli* (BL21 pLysS) was purified using Glutathione Sepharose high performance (GE healthcare). (DOCX 13 kb)

Additional file 8: **Table S1.** Primers used in this manuscript. (XLSX 12 kb)

## Abbreviations

3-AT: 3-amino-1, 2, 4-triazole
ABA: Abscisic acid
Arabidpsis: *Arabidopsis thaliana*
CIPK: CBL-INTERACTING PROTEIN KINASEs
CPK: CALCIUM-DEPENDENT PROTEIN KINASE
GORK: K^+^-outward-rectifying channel
GUS: β-glucuronidase
KAT1: K^+^-inward-rectifying channel
MS: Murashige and Skoog
OST1: OPEN STOMATA 1
PCR: Polymerase chain reaction
PEG: Polyethylene glycol electrolyte
PPT: Phosphinothricin
SD: Synthetic Defined
SLAC1: Slow-type anion channel
SnRK2: SNF1-RELATED PROTEIN KINASE2
