# Supplementary figures and images for: Two Clade A Phosphatase 2Cs Expressed in Guard Cells Physically Interact With Abscisic Acid Signaling Components to Induce Stomatal Closure in Rice
Source: Rice (N Y). 2019 May 27;12:37. doi: 10.1186/s12284-019-0297-7 (PMC6536566; doi:10.1186/s12284-019-0297-7)

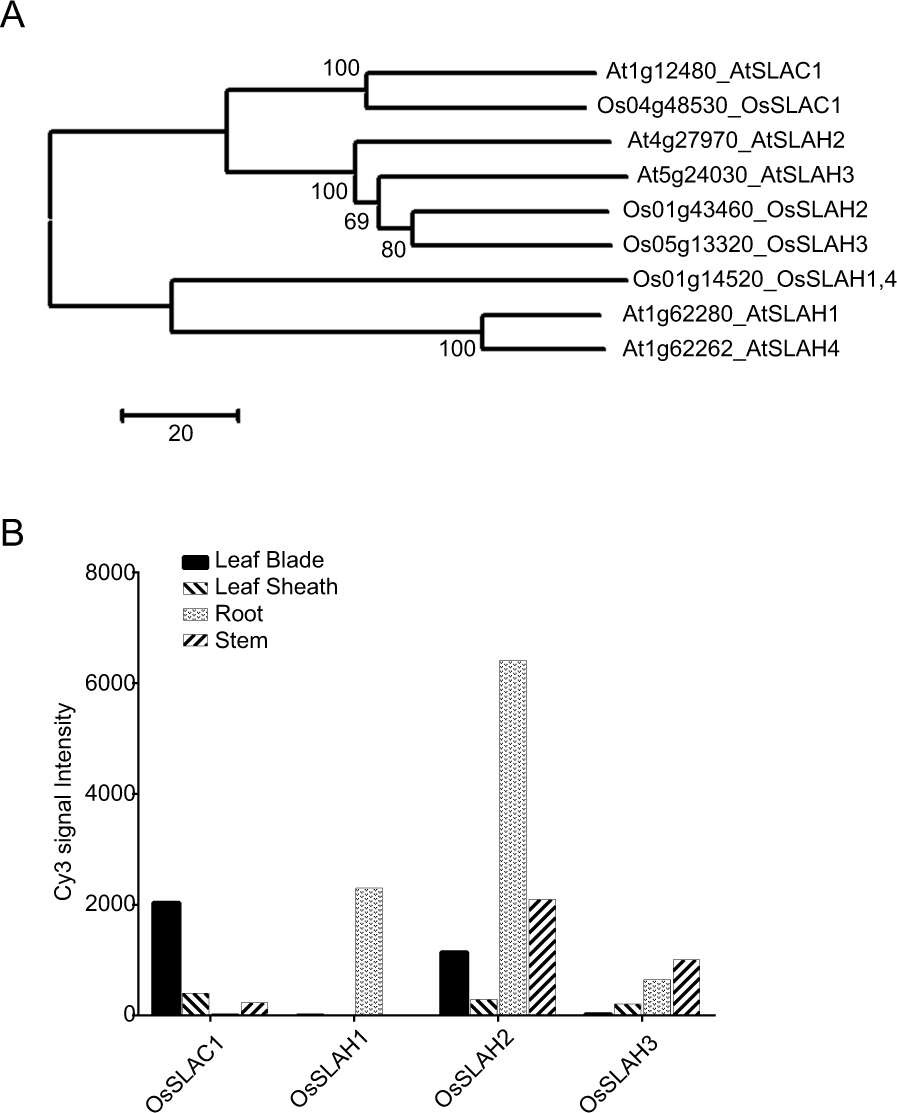

Supplement: Supplementary file 1 — Figure S1. OsSLAC1 has the highest homology to AtSLAC1 and is expressed predominantly in leaf tissues. (A) Phylogenetic tree depicting S-type anion channels of Arabidopsis and their homologues in rice. This tree was constructed using the neighbor-joining method in Mega 6.0. (B) Tissue-specific expression data for rice S-type anion channels were analyzed using the Rice X pro database. (TIF 3251 kb) [file 12284_2019_297_MOESM1_ESM.tif]

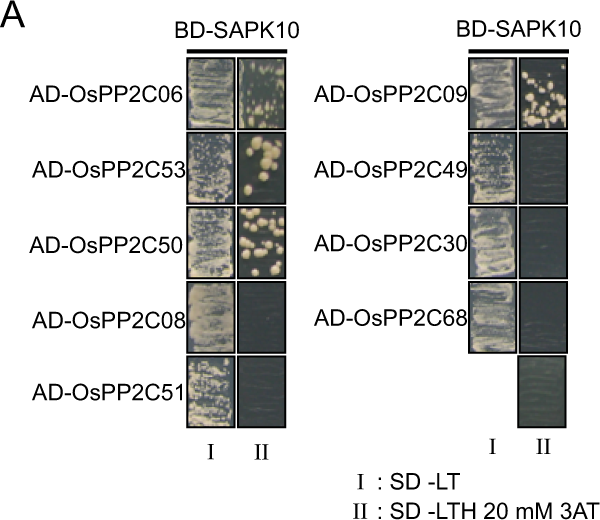

Supplement: Supplementary file 2 — Figure S2. Yeast two hybridization assay between BD-SAPK10 and AD-OsPP2CAs. SD: Synthetic Drop-out medium, L: leucine, T: Tryptophan, H: Histidine, 3AT: 3-amino-1,2,4-triazole. (TIF 1196 kb) [file 12284_2019_297_MOESM2_ESM.tif]

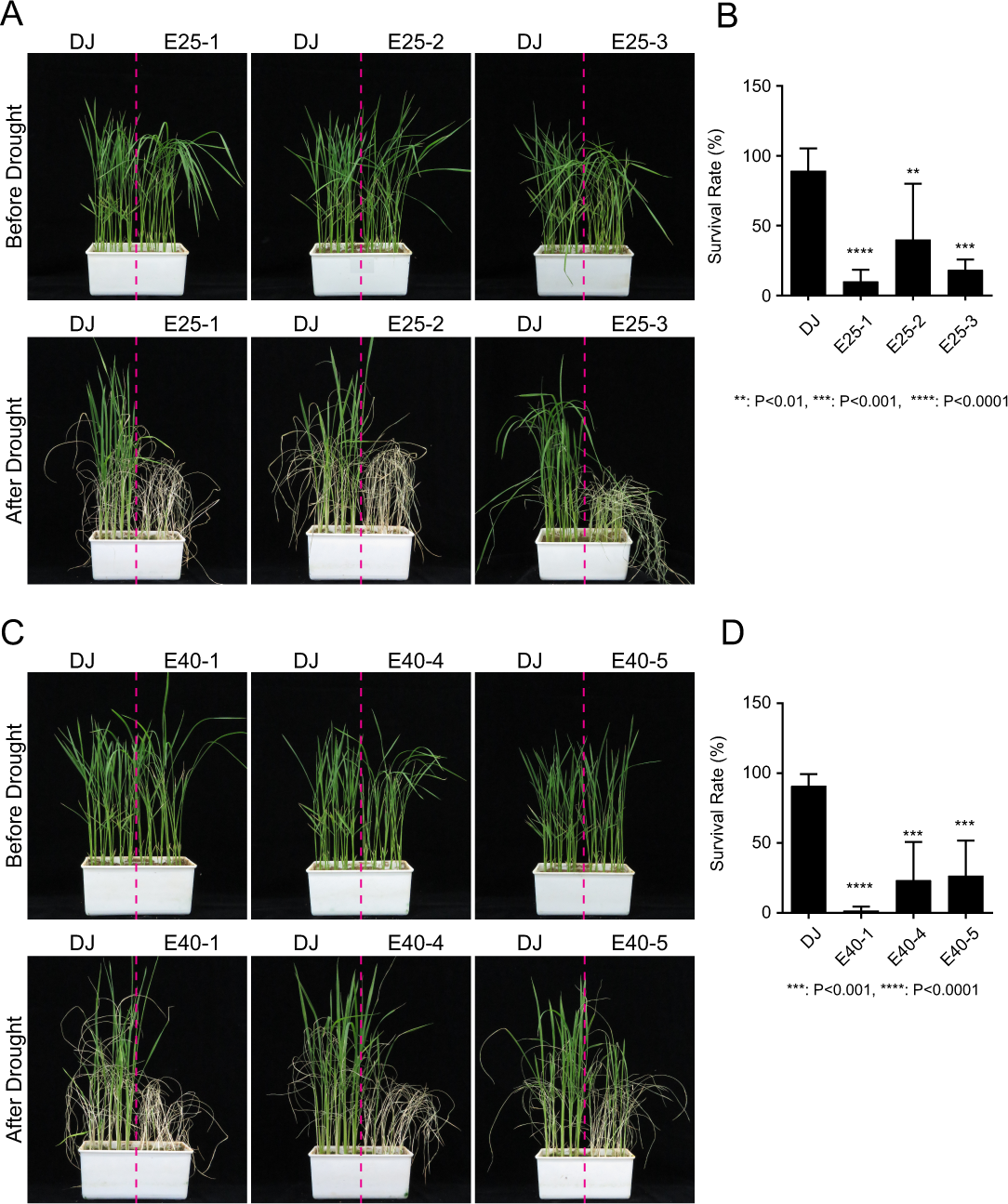

Supplement: Supplementary file 3 — Figure S3. Transgenic rice overexpressing OsPP2C50 and OsPP2C53 present drought hypersensitive phenotype. Pictures showing drought hypersensitivity of OsPP2C53-overexpressing transgenic lines (E25–1, 2 and 3) compared to control line (DJ, DongJin) (A) and OsPP2C50-overexpressing transgenic lines (E40–1, 4 and 5) compared to control line (C). (B, D) Survival rate after rewatering the drought treated rice of each transgenic line. These data obtained from 3 independent experiment, N > 20. The values depict the average and ± SD. (TIF 6522 kb) [file 12284_2019_297_MOESM3_ESM.tif]

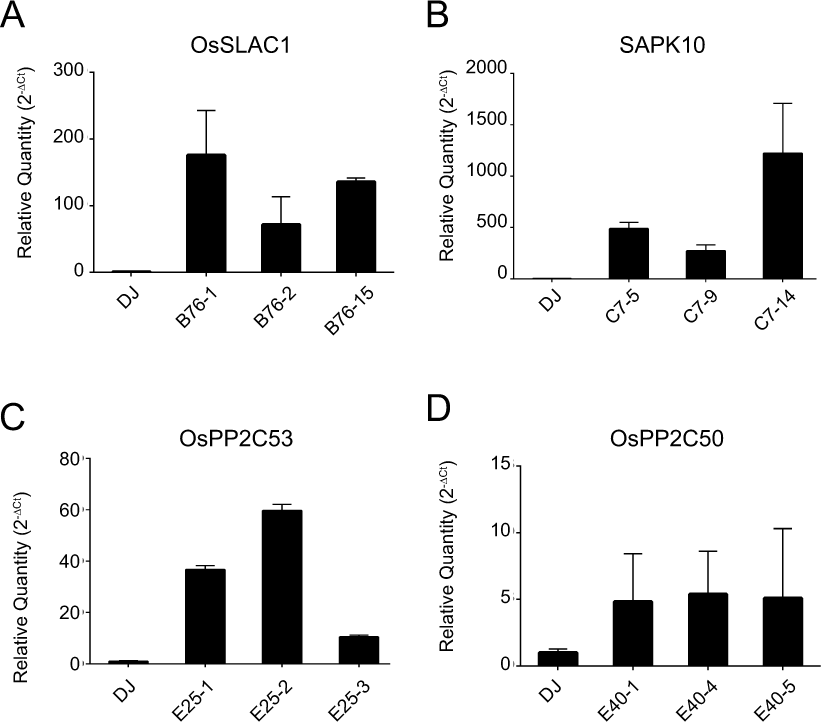

Supplement: Supplementary file 4 — Figure S4. Confirmation of gene overexpression in transgenic rice. All overexpressing transgenic plants were confirmed by RT-qPCR analysis with each specific primer. The expression level of Ubiqutin5 was used by endogenous control. All values were compared to the value of DJ. (A) OsSLAC1, (B) SAPK10, (C) OsPP2C53, and (D) OsPP2C50. (TIF 1919 kb) [file 12284_2019_297_MOESM4_ESM.tif]

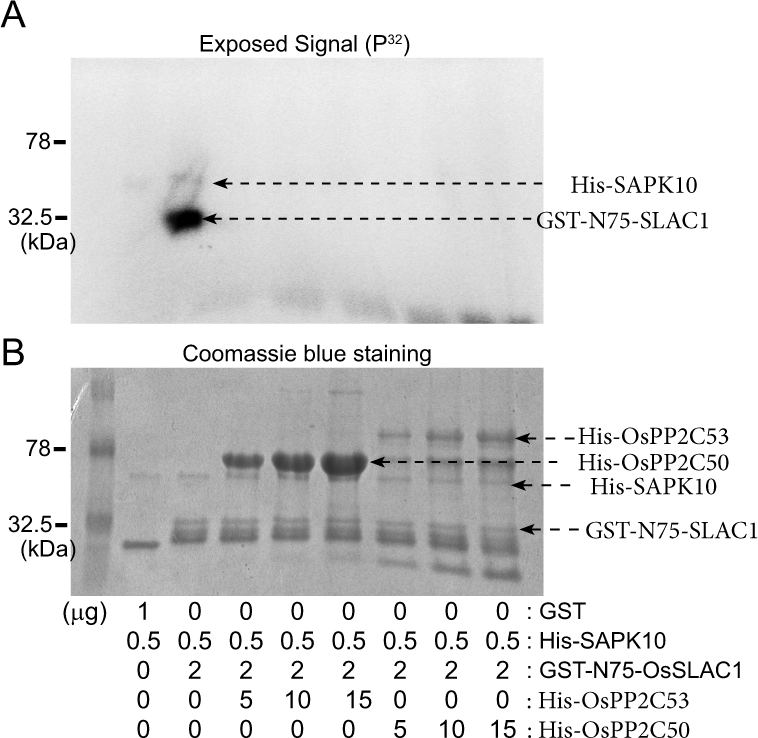

Supplement: Supplementary file 5 — Figure S5. Inhibition of kinase activity of SAPK10 by OsPP2C50 or OsPP2C53 In vitro kinase assay were performed with OsPP2C50 or OsPP2C53. Each protein were used indicated quantity (below) and phosphorylated with SAPK10 in reaction buffer (60 mM NaCl, 20 mM Tris-Cl, pH 7.4, 5 mM MgCl2, 10 μM ATP, and 10 μCi γ-32P-ATP) at 30 °C for 30 min. (A) Detected phosphor image, (B) Polyacrylamide gel stained with Coomassie brilliant blue R250. (TIF 2460 kb) [file 12284_2019_297_MOESM5_ESM.tif]

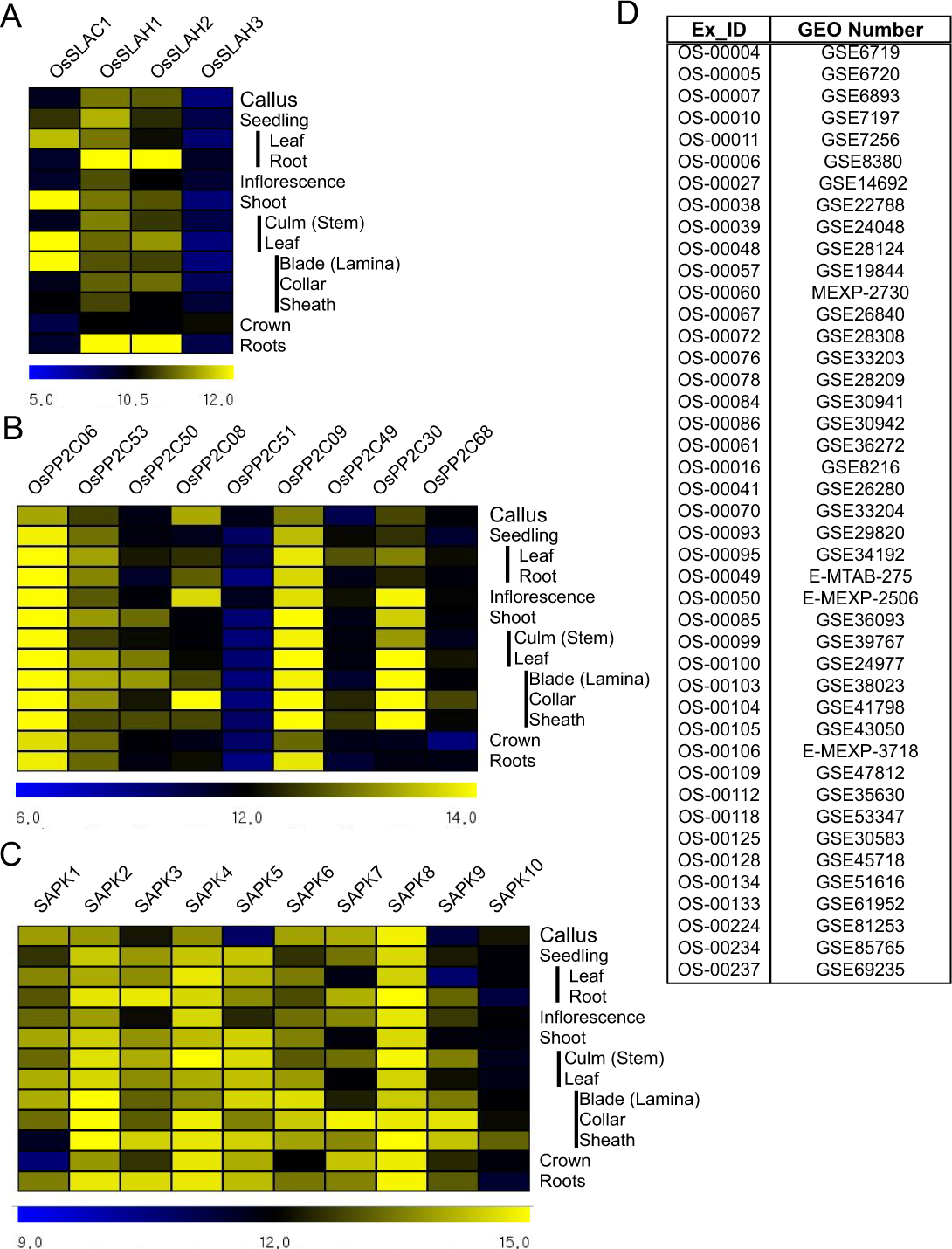

Supplement: Supplementary file 6 — Figure S6. The expression of SAPKs, OsPP2Cs and OsSLAC1/OsSLAHs in anatomy microarray data base. (A,B,C) OsSLAC1/OsSLAHs, OsPP2Cs, and SAPKs expression were search in microarray data base (Genevestigator). (D) The experimental ID (ex_ID) and used GEO numbers of DATA sets. (TIF 5518 kb) [file 12284_2019_297_MOESM6_ESM.tif]
