# Supplementary material for: Two Clade A Phosphatase 2Cs Expressed in Guard Cells Physically Interact With Abscisic Acid Signaling Components to Induce Stomatal Closure in Rice
Source: Rice (N Y). 2019 May 27;12:37. doi: 10.1186/s12284-019-0297-7 (PMC6536566; doi:10.1186/s12284-019-0297-7)
Supplement: Supplementary file 7 — Supplementary methods. Purification of GST-N75-OsSLAC1. To obtain the GST-N75-OsSLAC1, we constructed pGEX-5x-N75-OsSLAC1. N-terminal region (N75) of OsSLAC1 was amplified by PCR reaction with specific primers (Additional file 8: Table S1) and harbored in pGEX-5x-1 vector with digestion by restriction enzymes (BamH1 and EcoR1) and ligation. Expressed GST-N75-OsSLAC1 in E. coli (BL21 pLysS) was purified using Glutathione Sepharose high performance (GE healthcare). (DOCX 13 kb) [file 12284_2019_297_MOESM7_ESM.docx]

**Supplementary Methods**

**Purification of GST-N75-OsSLAC1**

To obtain the GST-N75-OsSLAC1, we constructed *pGEX-5x-N75-OsSLAC1*. N-terminal region (N75) of OsSLAC1 was amplified by PCR reaction with specific primers (supplementary table fv) and harbored in pGEX-5x-1 vector with digestion by restriction enzymes (BamH1 and EcoR1) and ligation. Expressed GST-N75-OsSLAC1 in E. Coli (BL21 pLysS) was purified using Glutathione Sepharose high performance (GE healthcare).
